# EGFR phosphorylates HDAC1 to regulate its expression and anti-apoptotic function

**DOI:** 10.1038/s41419-021-03697-6

**Published:** 2021-05-11

**Authors:** Sonali Bahl, Hongbo Ling, Nuwan P. N. Acharige, Irene Santos-Barriopedro, Mary Kay H. Pflum, Edward Seto

**Affiliations:** 1 Department of Biochemistry & Molecular Medicine, The George Washington University School of Medicine & Health Sciences, Washington, DC USA; 2 GW Cancer Center, The George Washington University School of Medicine & Health Sciences, Washington, DC USA; 3 Department of Chemistry, Wayne State University, Detroit, MI USA

**Keywords:** Chemical modification, Phosphorylation

## Abstract

HDAC1 is the prototypical human histone deacetylase (HDAC) enzyme responsible for catalyzing the removal of acetyl group from lysine residues on many substrate proteins. By deacetylating histones and non-histone proteins, HDAC1 has a profound effect on the regulation of gene transcription and many processes related to cell growth and cell death, including cell cycle progression, DNA repair, and apoptosis. Early studies reveal that, like most eukaryotic proteins, the functions and activities of HDAC1 are regulated by post-translational modifications. For example, serine phosphorylation of HDAC1 by protein kinase CK2 promotes HDAC1 deacetylase enzymatic activity and alters its interactions with proteins in corepressor complexes. Here, we describe an alternative signaling pathway by which HDAC1 activities are regulated. Specifically, we discover that EGFR activity promotes the tyrosine phosphorylation of HDAC1, which is necessary for its protein stability. A key EGFR phosphorylation site on HDAC1, Tyr72, mediates HDAC1’s anti-apoptotic function. Given that HDAC1 overexpression and EGFR activity are strongly related with tumor progression and cancer cell survival, HDAC1 tyrosine phosphorylation may present a possible target to manipulate HDAC1 protein levels in future potential cancer treatment strategies.

## Introduction

Histone deacetylases (HDACs) are enzymes that are best-known to catalyze the removal of acetylation from lysine residues within their target substrates. The human HDAC family consists of 18 members that are classified according to homology to yeast deacetylases into the Classical HDACs and the Sirtuins^1^. HDAC1, a member of the Class I HDACs, was first discovered in biochemical copurification experiments with the irreversible HDAC activity inhibitor trapoxin and possesses significant sequence homology to the yeast Rpd3 protein^2^. Compared to many other HDACs, HDAC1 has measurable robust deacetylase activity and significantly contributes to the total cellular deacetylase activity^3,4^.

HDAC1 is an important factor involved in many mechanisms that regulate cell growth and survival^3,5–8^. In regards to the regulation of apoptosis, in particular, HDAC1 plays different context-dependent roles. Downstream of stimulation by transforming growth factor-β1 (TGF-β1), HDAC1 promotes apoptosis through inhibition of the phosphorylation and activation of extracellular signal-regulated kinases 1/2 (ERK1/2) in mouse hepatocytes^9^. However, loss of Rpd3, which is orthologous to HDAC1 and HDAC2, in *Drosophila* epithelial cells is associated with apoptosis through activation of the c-Jun N-terminal kinase (JNK) pathway and inhibition of Yorkie (Yki) transcriptional activity downstream of the Hippo pathway^10^. Lower HDAC1 expression owing to the reduced expression of Rhotekin (RTKN) in gastric cancer cells is proposed to lead to higher p53 acetylation levels, which allows for the increased expression of its target genes involved in promoting cell cycle arrest and apoptosis^11^. Due to the close involvement of HDAC1 in regulating cell fate, abnormal expression and activity of HDAC1 have been implicated in cancers. For instance, reduction in *HDAC1* expression has also been associated with apoptosis in glioblastoma cells^12^. However, loss of both *HDAC1* and *HDAC2* induces apoptosis in mouse oocytes^13^ and the SW579 thyroid cancer cell line^14^, whereas the absence of each gene individually has little effect likely due to compensation from the other. More interestingly, in lung adenocarcinoma, a histological subtype of NSCLC, high HDAC1 expression has been correlated with poor prognosis, a lower differentiation grade, and reduced five-year disease-free survival rate^15–18^. HDAC1 expression, specifically, in A549 NSCLC cells was associated with resistance to chemotherapy. Consistently, knockdown of HDAC1 expression sensitized resistant cells to paclitaxel treatment^18^.

The expression and activities of HDACs are regulated at multiple levels^19^. HDAC1 can be regulated by its interactions with other proteins and a variety of post-translational modifications (PTMs)^4^. Among them, HDAC1 regulation by serine phosphorylation has been best described^20–22^. Additional phosphorylation sites in HDAC1, of which several are tyrosine residues, have been detected by high-throughput mass spectrometry analyses but have not been validated to date; nothing further is known about these modifications and their effects on HDAC1 protein characteristics and functions^23^. Since tyrosine phosphorylation is involved in cellular signaling consequent of growth factor stimulation and has been related with cellular transformation and aberrant cell proliferation in cancer^24^, it is of clinical importance to evaluate new sites of tyrosine phosphorylation and their corresponding kinases, which may serve as potential therapeutic targets.

In the present study, we evaluate the modification of HDAC1 by tyrosine phosphorylation and its interaction with, and modulation by, EGFR in lung adenocarcinoma cells. We demonstrate that inhibition of EGFR kinase activity reduces the tyrosine phosphorylation and protein levels of HDAC1 in Gefitinib-sensitive lung adenocarcinoma cells. The effect of mutating HDAC1 at the putative phosphorylation site Tyr72 to the nonphosphorylatable phenylalanine (Y72F) is a reduction in HDAC1 levels and sensitivity to apoptosis corresponding to the effect observed upon EGFR inhibition. Whereas overexpression of WT HDAC1 promotes resistance against Gefitinib-induced apoptosis, overexpression of the nonphosphorylatable Y72F mutant reduces this effect. In totality, the findings of this study support the hypothesis that HDAC1 is regulated by EGFR activity and is likely phosphorylated at Tyr72, presenting a new possible target for therapeutic approaches to manipulate HDAC1.

## Results

### Tyrosine phosphorylation of HDAC1 is critical for the expression of HDAC1 protein

**Fig. 1:**
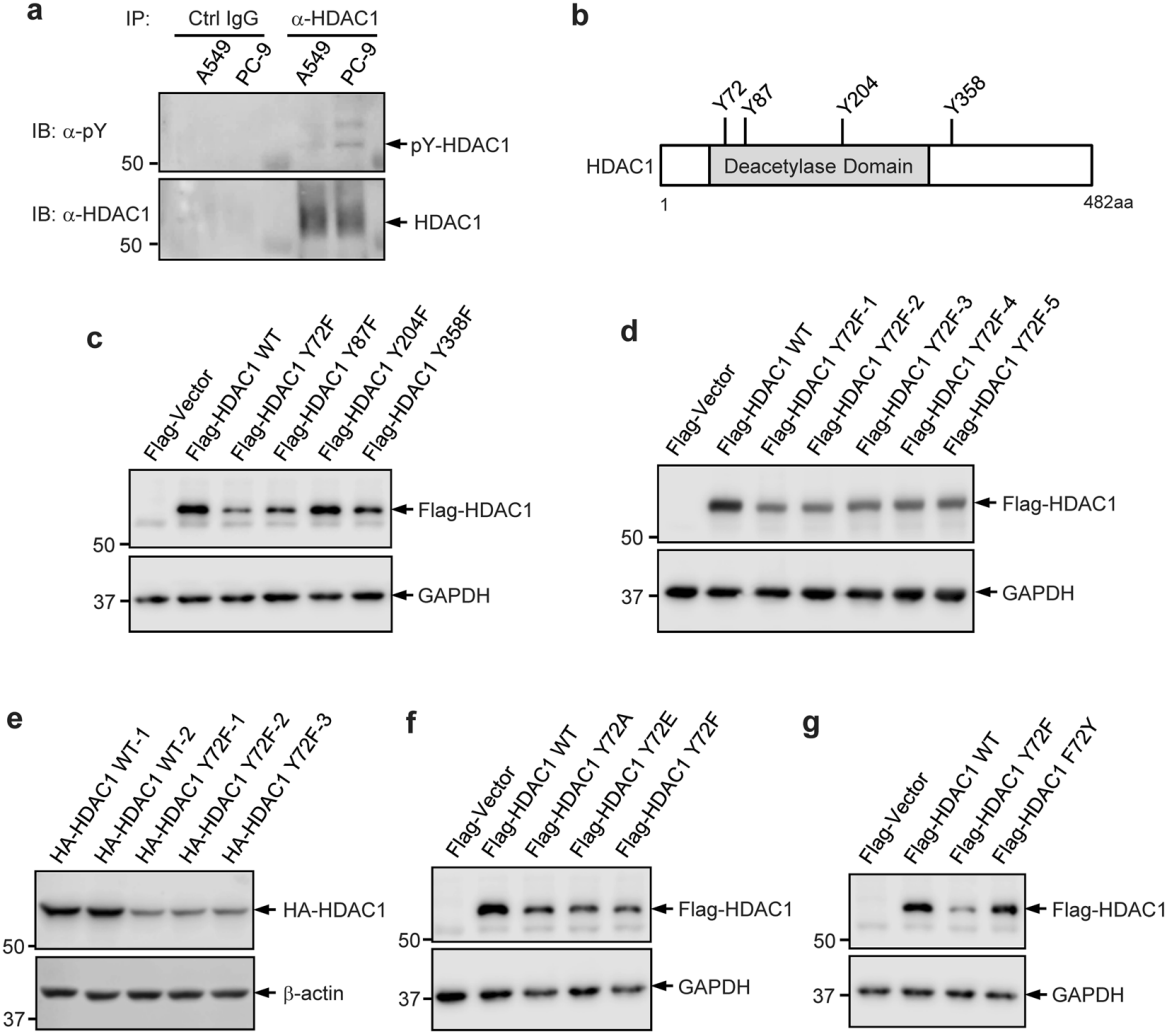
Phosphorylation of HDAC1 at Tyr72 is critical for the expression of HDAC1 protein. **a** Immunoprecipitation of endogenous HDAC1 and Western blot analysis to detect tyrosine phosphorylation in A549 and PC-9 cells with the indicated antibodies. **b** Predicted tyrosine phosphorylation sites in HDAC1 detected in non-small cell lung cancer cells. **c** Predicted tyrosine phosphorylation sites were mutated to the nonphosphorylatable phenylalanine (YF mutation). Plasmids were transiently transfected into HEK293T cells and their expression was detected by Western blot analysis. **d** Expression levels of Flag HDAC1 Y72F from several independent clones were assessed by Western blot. **e** HDAC1 WT and Y72F sequences were subcloned from the Flag-tag vector to an HA-tag vector. Plasmids were transiently transfected into HEK293T cells and their expression was detected by Western blot. **f** Flag HDAC1 plasmid was mutated at Tyr72 to alanine (A), glutamic acid (E), or phenylalanine (F). Plasmids were transiently transfected into HEK293T cells and their expression was detected by Western blot. **g** Flag HDAC1 Y72F mutant plasmid was mutated to express F72Y. Plasmids were transiently transfected into HEK293T cells and their expression was detected by Western blot.

To determine if HDAC1 undergoes tyrosine phosphorylation in lung adenocarcinoma cells, endogenous HDAC1 was immunoprecipitated from A549 and PC-9 cell lysates, and examined for the levels of phosphotyrosine by immunoblotting (Fig. 1a). Tyrosine phosphorylation of HDAC1 could be detected with a faint signal in A549 cells, and more visibly in PC-9 cells.

Mass spectrometry analysis revealed that four tyrosine residues (Tyr72, Tyr87, Tyr204, and Tyr358) were putatively phosphorylated in the PC-9 lung adenocarcinoma cell line^25^ (Fig. 1b). We next evaluated the potential role of these putative phosphorylation sites by generating individual tyrosine-to-phenylalanine (YF) mutations in a Flag-tagged HDAC1 plasmid to mimic hypophosphorylation. These mutant plasmids were transiently transfected into HEK293T cells and their relative expression was examined (Fig. 1c). Whereas HDAC1 expression was unchanged upon mutation of Tyr204, it was reduced to varying extents upon mutation of Tyr72, Tyr87, or Tyr358. Specifically, mutation of Tyr72 resulted in the largest decrease in the expression of HDAC1 protein. The effect of the individual mutation of Tyr72 on HDAC1 protein expression was further evaluated with the following several strategies. Independent Flag-tagged Y72F mutant HDAC1 clones were transfected into HEK293T cells and their expression relative to WT HDAC1 was compared. The reduction in HDAC1 protein expression was consistent among the different mutant clones (Fig. 1d). Additionally, WT and Y72F mutant HDAC1 were subcloned into the HA-tag vector and the plasmids were transfected into HEK293T cells. The expression of HA-tagged HDAC1 was determined (Fig. 1e). Consistently, the expression of the HA-tagged Y72F mutant HDAC1 remained lower than that of HA-tagged WT HDAC1, indicating that the observed effect was not due to an unintentional issue that occurred in the plasmid following mutagenesis. The Tyr72 residue was replaced with some other amino acids, besides phenylalanine, and the expression of HDAC1 mutant was examined. Mutation of Tyr72 to alanine (A), another common loss-of-phosphorylation mimic, similarly resulted in reduced HDAC1 expression relative to WT HDAC1 (Fig. 1f). Though there is no established mimic for the constitutive phosphorylation of tyrosine, glutamic acid (E) is one amino acid that is often used to mimic serine phosphorylation. The Y72E mutant HDAC1 was generated to test whether this mutation could possibly lead to the hypothesized increase in HDAC1 expression. The Y72E mutation, however, was also associated with lower levels of HDAC1, suggesting that this mutation is not likely to mimic the constitutive phosphorylation of HDAC1 at Tyr72. To further support this phenotype and eliminate the possibility that the observed effect was due to an error during the plasmid mutation, a reverse phenylalanine-to-tyrosine mutation (F72Y) was generated using the Y72F plasmid as the template. Mutation of the Y72F mutant to F72Y, which is theoretically once again WT HDAC1, successfully rescued HDAC1 protein expression (Fig. 1g). Collectively, these evaluations indicate that the Tyr72 residue and, theoretically, its phosphorylation are critical to maintain HDAC1 expression.

### EGFR interacts with HDAC1 in a kinase-activity-independent manner

**Fig. 2:**
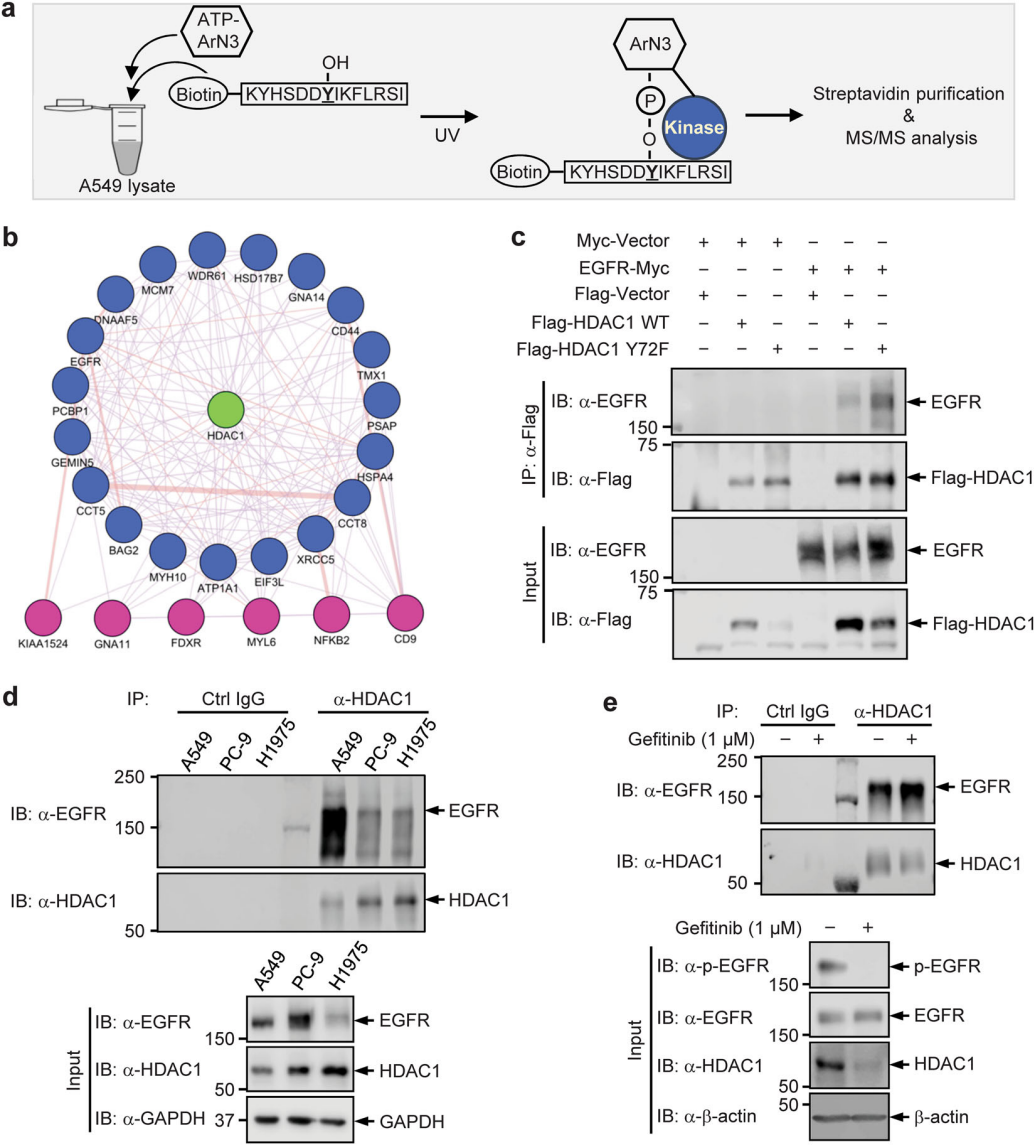
EGFR interacts with HDAC1 in a kinase-activity-independent manner. **a** Illustration of K-CLASP technique to identify the interactome of Tyr72-containing peptide. **b** Interactome analysis of proteins in A549 cell lysate predicted to interact with HDAC1 with high-confidence by the K-CLASP technique. **c** Identification of the interaction of Flag-tagged HDAC1 with Myc-tagged EGFR. HEK293T cells were co-expressed with Flag-tagged HDAC1 and Myc-tagged EGFR or control. Cell lysates were subjected to immunoprecipitation with an antibody to Flag tag and blotted with the indicated antibodies. **d** Endogenous HDAC1 was immunoprecipitated from A549, PC-9, and H1975 cell lysates and its interaction with EGFR was detected by Western blot. **e** HDAC1 interaction with EGFR was assessed following treatment of PC-9 cells with 1 μM Gefitinib for 24 h.

We next asked which tyrosine kinase(s) is responsible for the tyrosine phosphorylation of HDAC1. Particularly, since the phosphorylation at Tyr72 is important for the expression of HDAC1 protein, we sought to identify the tyrosine kinase that potentially phosphorylates HDAC1 at Tyr72 using the K-CLASP assay. K-CLASP is an approach by which kinases that modify a protein at a specific phosphorylation site, as well as interacting proteins of potential functional relevance, can be discovered^26^. This strategy offers the ability to identify transient interactions or occurrences at low abundance which may be difficult to capture otherwise. In this assay, A549 cell lysate was incubated with a biotinylated HDAC1 WT peptide containing the sequence surrounding Tyr72 and ATP-arylazide as a photocrosslinker under ultraviolet (UV) radiation. Proteins present in the lysate, including kinases, that interacted with the peptide were detected and identified by mass spectrometry (Fig. 2a). Negative controls included incubation of the lysate: 1) with the WT peptide and ATP-arylazide but without UV; 2) with the WT peptide, ATP, and UV; and 3) with UV, ATP-arylazide, and the HDAC1 Y72A peptide instead of the WT peptide. Using a cut-off of 1.5-fold enrichment relative to negative controls for a high confidence prediction, 25 proteins present in the A549 cell lysate interacted with the HDAC1 WT peptide, of which EGFR is the only kinase (Fig. 2b, Supplementary Tables S1 and S2).

We next further identified the potential EGFR-HDAC1 interaction. Cells were co-transfected with plasmids expressing EGFR and Flag-tagged HDAC1 WT or Y72F mutant, and subjected to immunoprecipitation with an anti-Flag antibody. Although the EGFR-HDAC1 interaction was initially identified in the K-CLASP assay with a Tyr72-containing WT peptide, both Flag-tagged HDAC1 WT and Y72F proteins were readily co-immunoprecipitated with EGFR protein (Fig. 2c). Thus, it is likely that EGFR can phosphorylate some tyrosine residues other than Tyr72 of HDAC1. Consistent with this, the endogenous interaction between HDAC1 and EGFR was detected in all tested cell lines (A549, PC-9 and H1975) (Fig. 2d). Interestingly, the interaction was stronger in A549 cells (expressing WT EGFR) relative to PC-9 and H1975 cells (both expressing constitutively-active EGFR mutant), implying that the EGFR-HDAC1 interaction does not rely on the kinase activity of EGFR. If such is the case, inhibition of EGFR kinase activity should not disrupt the interaction of HDAC1 with EGFR. To test this hypothesis, PC-9 cells, which are sensitive to the EGFR tyrosine kinase inhibitor (TKI) treatment, were treated with 1 μM TKI Gefitinib, and then subjected to co-immunoprecipitation with the anti-HDAC1 antibody. While it diminished the tyrosine phosphorylation of EGFR, the Gefitinib treatment did not disrupt the HDAC1-EGFR interaction (Fig. 2e). Thus, EGFR interacts with HDAC1 in a manner independent of its kinase activity.

### EGFR phosphorylates HDAC1

**Fig. 3:**
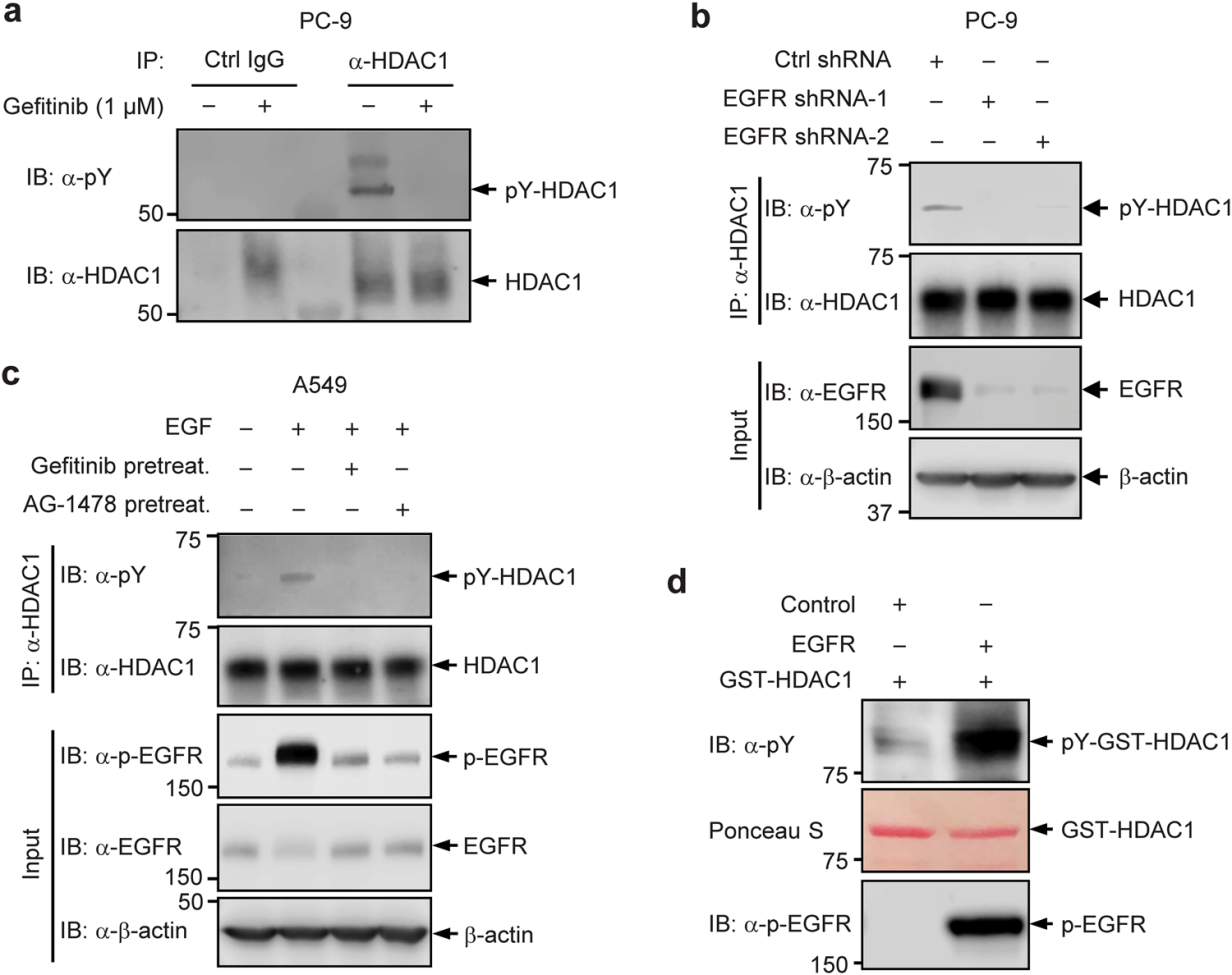
EGFR phosphorylates HDAC1. **a** Tyrosine phosphorylation of HDAC1 in PC-9 cells without and with 1 μM Gefitinib treatment for 24 h. **b** PC-9 cells were transduced with lentivirus expressing either control shRNA (shCtrl) or shRNAs targeting *EGFR*. HDAC1 protein was immunoprecipitated and examined for the tyrosine phosphorylation. **c** A549 cells were pre-treated with 1 µM Gefitinib, 1 µM AG-1478, or control. Cells were then stimulated with 20 ng/ml EGF for 30 min. HDAC1 protein was immunoprecipitated and examined for tyrosine phosphorylation. **d** H1299 cells were transfected with Flag-tagged EGFR or control, and then stimulated with EGF. Active EGFR or control was immunoprecipitated from cells, and subjected to in vitro kinase assay with bacterially purified GST-tagged HDAC1. The reaction mixtures were probed with the indicated antibodies.

Having shown EGFR interaction with HDAC1, we examined if EGFR phosphorylates HDAC1. We first tested if EGFR inhibition will attenuate the tyrosine phosphorylation level of HDAC1 protein. PC-9 cells, which harbor constitutively active EGFR mutant and are sensitive to the treatment of Gefitinib, a first-generation EGFR TKI that targets the tyrosine kinase domain of EGFR in its active conformation^27,28^, were employed in this assay. PC-9 cells were treated with Gefitinib. The endogenous HDAC1 was immunoprecipitated with the anti-HDAC1 antibody and examined for the tyrosine phosphorylation level. Whereas tyrosine phosphorylation of HDAC1 could be detected in untreated PC-9 cells, this modification was abolished in Gefitinib-treated cells (Fig. 3a), suggesting that the modification is likely dependent on EGFR activity. Consistently, shRNA-mediated depletion of EGFR protein in PC-9 cells led to a reduction in the tyrosine phosphorylation of HDAC1 (Fig. 3b). Thus, EGFR inhibition blocks the tyrosine phosphorylation of HDAC1.

We next tested if the activation of EGFR will enhance the tyrosine phosphorylation level of HDAC1. To this end, A549 cells were starved, followed by exposure to TKI (Gefitinib or AG-1478) or control. Cells were then stimulated with EGF, and the tyrosine phosphorylation levels of HDAC1 were determined (Fig. 3c). The EGF stimulation remarkably activated EGFR, resulting in an increase in the tyrosine phosphorylation of HDAC1. When EGFR activity was inhibited by either Gefitinib or AG-1478, the EGF-induced enhancement of tyrosine phosphorylation of HDAC1 was largely attenuated, implying that EGFR phosphorylates HDAC1. We next performed an in vitro kinase assay, as described previously^29^, to test if EGFR directly phosphorylates HDAC1 in vitro. Bacterially purified GST-tagged HDAC1 was incubated with EGF-activated EGFR in a kinase buffer. Activated EGFR remarkably increased the tyrosine phosphorylation level of GST-tagged HDAC1 (Fig. 3d).

### EGFR inhibition destabilizes HDAC1 protein by promoting its ubiquitination

**Fig. 4:**
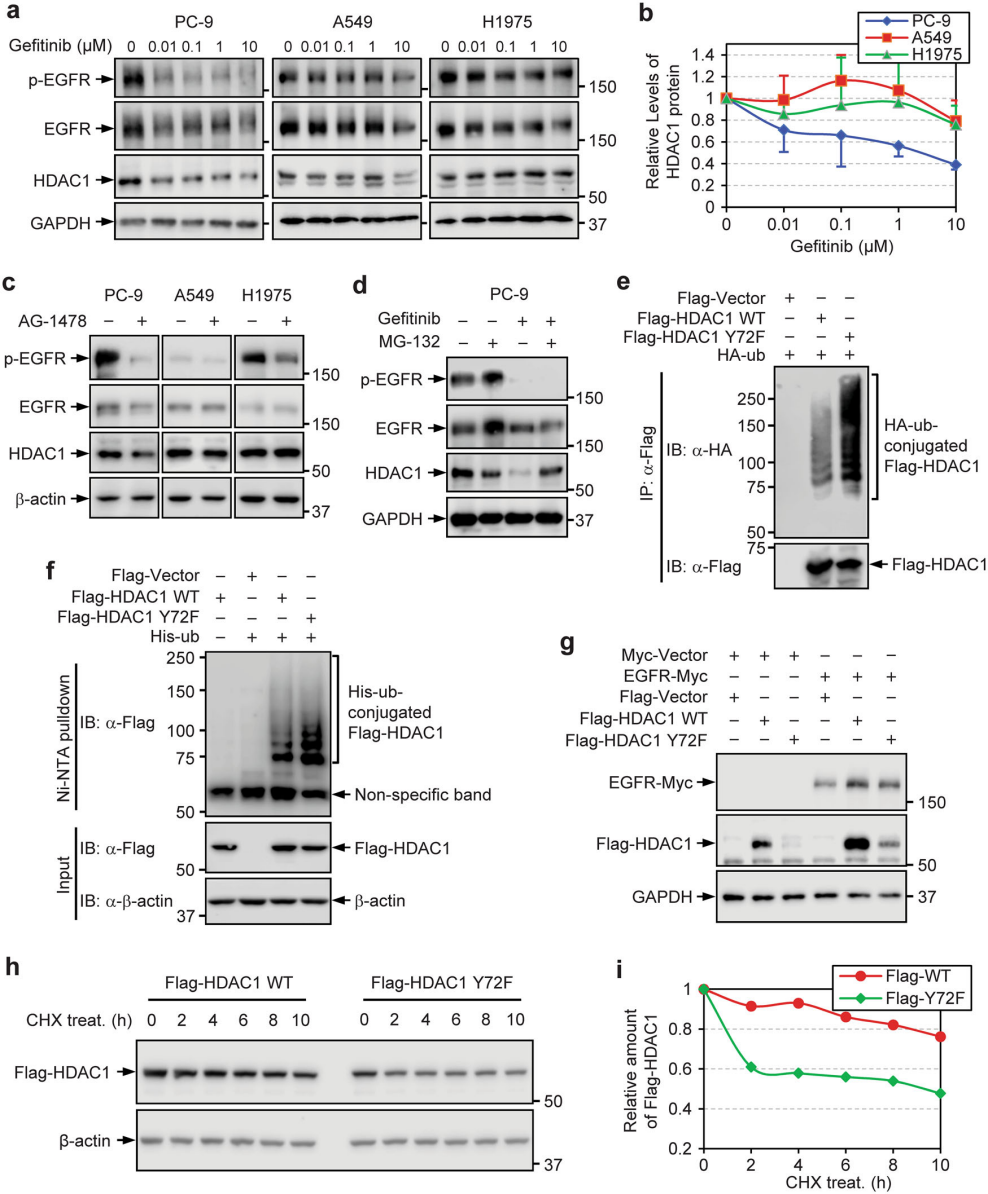
EGFR inhibition destabilizes HDAC1 protein by promoting its ubiquitination. **a** PC-9, A549 and H1975 cells were treated with the indicated dose of Gefitinib for 24 h and HDAC1 expression was assessed by Western blot analysis. **b** Quantification of HDAC1 expression following treatment with the indicated dose of Gefitinib. Graphed values indicate mean ± SEM, *n* = 4 for PC-9 cells or n = 3 for A549 and H1975 cells. **c** PC-9, A549 and H1975 cells were treated with 1 µM AG-1478 for 24 h. HDAC1 expression was assessed by Western blot. **d** PC-9 cells were treated with 1 μM Gefitinib for 24 h and 10 μM MG-132 4 h prior to collection and HDAC1 expression was assessed by Western blot analysis. **e** HEK293T cells were co-transfected with plasmids expressing HA-tagged ubiquitin and Flag-tagged HDAC1, and were treated with MG-132. Cell lysates were precipitated with an anti-Flag tag antibody and blotted with an anti-HA tag antibody. **f** HEK293T cells were co-transfected with plasmids expressing His_6_-tagged ubiquitin and Flag-tagged HDAC1, and were treated with MG-132. Cell lysates were subjected to pulldown with Ni-NTA agarose, and blotted with an antibody to Flag. **g** Myc-tagged EGFR and either Flag-tagged HDAC1 WT or Y72F mutant were co-expressed in HEK293T cells and were analyzed by Western blot. **h** HEK293T cells were transfected with plasmids expressing Flag-tagged HDAC1 WT or Y72F mutant. Cells were exposed to 100 µM cycloheximide (CHX) for up to 10 h. At every 2 h, cell lysates were collected and the expression of Flag-tagged HDAC1 was assessed. **i** Quantification of the expression of Flag-tagged HDAC1.

EGFR activating mutations cause oncogene addiction in non-small cell lung cancer^30–32^. Our initial results have indicated that the tyrosine phosphorylation of HDAC1 is important for the expression of HDAC1 protein (Fig. 1c–g). Given that EGFR is the chief kinase that phosphorylates the tyrosine residues of HDAC1, it is important to characterize how EGFR activity can regulate HDAC1 expression. PC-9, A549 and H1975 cells were treated with Gefitinib. HDAC1 expression was examined by immunoblotting (Fig. 4a) and its relative level was quantified (Fig. 4b). HDAC1 expression was reduced upon Gefitinib treatment in the Gefitinib sensitive PC-9 cells. A reduction in HDAC1 expression could be noted at a dose as low as 0.01 μM Gefitinib. In contrast, in A549 cells and H1975 cells, which are not as responsive to Gefitinib, HDAC1 protein levels were not significantly affected. When cells were treated with another TKI AG-1478, similar results were observed (Fig. 4c). Compellingly, loss of EGFR activity decreases the expression of HDAC1 protein.

HDAC1 has been shown to undergo ubiquitin-proteasome-dependent degradation^33,34^. It is likely that the effect of EGFR inhibition on HDAC1 expression relies on the ubiquitin-proteasome-dependent degradation. To address this, PC-9 cells were treated with Gefitinib and/or proteasome inhibitor MG-132, or control. The expression of HDAC1 protein was determined (Fig. 4d). Without the Gefitinib pre-treatment, the relative expression of HDAC1 might be slightly changed due to the proteasome inhibition. In contrast, when cells were pre-treated with Gefitinib, the loss of HDAC1 following Gefitinib treatment of PC-9 cells could be largely rescued by proteasome inhibition. Thus, the decrease in the HDAC1 expression caused by the EGFR inhibition is likely due to increased ubiquitin-proteasome-dependent degradation. In this context, the nonphosphorylatable mutation, like Y72F, may facilitate HDAC1’s ubiquitination. To test this hypothesis, HEK293T cells were co-expressed with HA-tagged ubiquitin and Flag-tagged HDAC1 WT or Y72F mutant. Cells were then treated with proteasome inhibitor MG-132 and the ubiquitination levels of Flag-tagged HDAC1 were examined. As expected, the ubiquitination levels of the Y72F mutant is ~2.6-fold higher than that of WT (Fig. 4e). In parallel, HEK293T cells were co-transfected with plasmids expressing His_6_-tagged ubiquitin and Flag-tagged HDAC1 WT or Y72F, and were exposed to MG-132. Cell lysates were subjected to pulldown with Ni-NTA beads, as described previously^35^, and were probed for His_6_-tagged ubiquitin-conjugated HDAC1 with an antibody to Flag tag (Fig. 4f). The Y72F mutation elevated the level of His_6_-tagged ubiquitin-conjugated HDAC1, implying that active EGFR-mediated phosphorylation protects HDAC1 from being ubiquitinated.

EGFR overexpression is associated with its increased activation^31,36^, and may therefore influence HDAC1 stability. To test this, HEK293T cells were co-transfected with plasmids expressing Myc-tagged EGFR and either Flag-tagged WT or Y72F mutant HDAC1. Co-expression of EGFR resulted in increased protein expression of WT HDAC1. Interestingly, the expression of the Y72F mutant also increased slightly upon co-expression of EGFR, though its protein levels still remained less than WT HDAC1 (Fig. 4g). These results suggest that EGFR may regulate HDAC1 stability at least partially through Tyr72. We next tested whether Tyr72 mutation affects the stability of HDAC1. Cells were transfected with plasmids expressing Flag-tagged HDAC1 WT or Y72F mutant, and were exposed to cycloheximide (CHX) for 2 h to 10 h. The Flag-tagged HDAC1 proteins were examined by immunoblotting (Fig. 4h) and their relative levels were quantified (Fig. 4i) to determine the half-life (t_1/2_) of the respective WT and mutant HDAC1. The 2 h CHX treatment, which had little effect on the expression of the WT protein, reduced the level of the Y72F mutant to ~60%. The t_1/2_ of the Y72F mutant was less than 10 h, whereas the t_1/2_ of the WT protein extended to more than 10 h, supporting the notion that the Tyr72 phosphorylation is an important determinant for the stability of HDAC1 protein.

### EGFR-mediated blockage of nuclear export of HDAC1 contributes to stabilizing HDAC1

Since HDAC1 is well-established as a nuclear protein, whereas the receptor tyrosine kinase EGFR is classically considered to be localized to the plasma membrane^1,37^, how EGFR interacts with and phosphorylates HDAC1 remains to be addressed. Recently, there have been some instances described in which HDAC1 can be localized to the cytoplasm or other cellular organelles, such as the endoplasmic reticulum^38–40^. Additionally, multiple studies have demonstrated that EGFR can be trafficked to the nucleus^41–44^, supporting that the EGFR-HDAC1 interaction is possible.

**Fig. 5:**
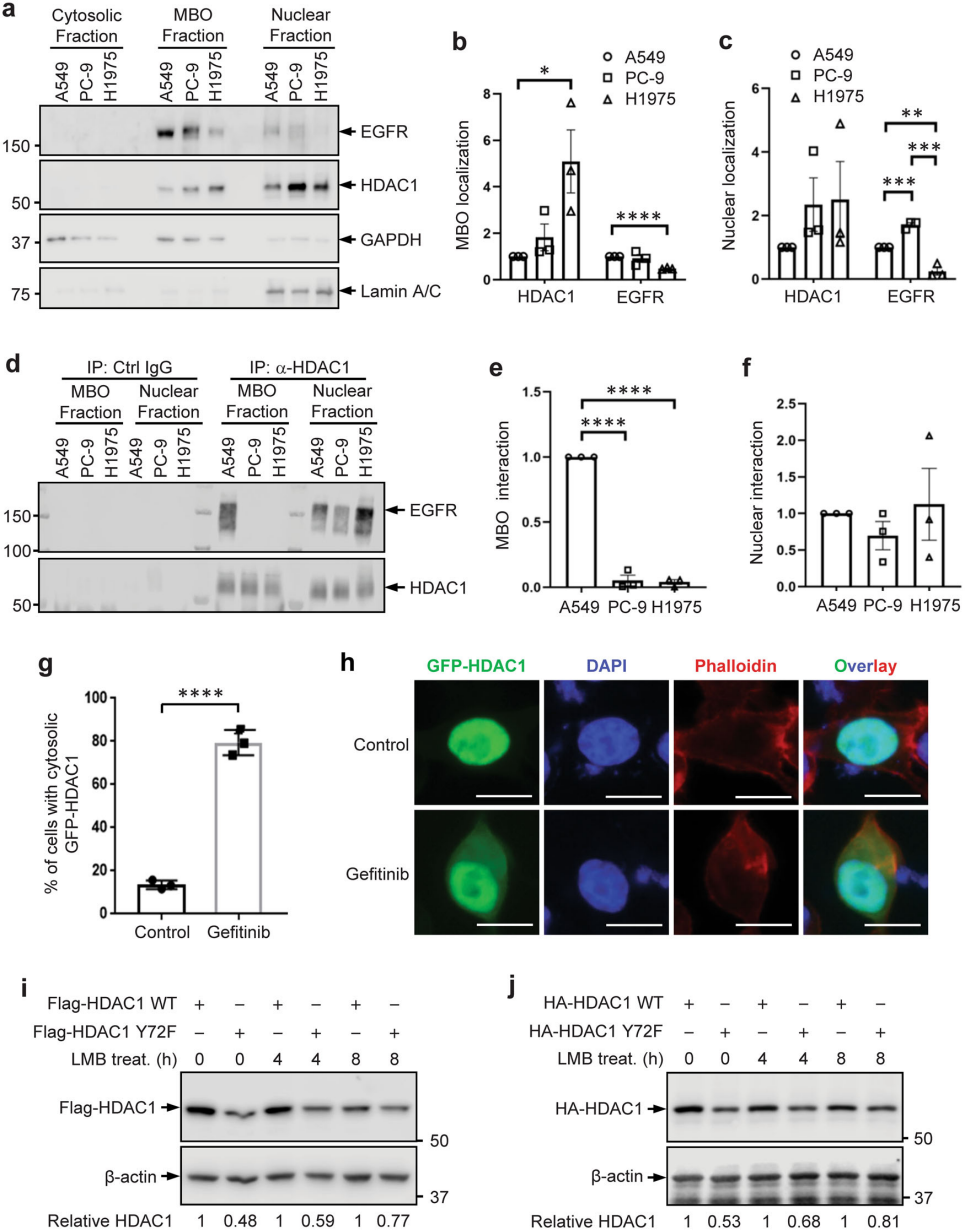
EGFR-mediated blockage of nuclear export of HDAC1 contributes to stabilizing HDAC1. **a** Presence of EGFR and HDAC1 in the cytosolic, membrane-bound organelle (MBO), and nuclear fractions of A549, PC-9, and H1975 cells was assessed by subcellular fractionation. Quantification of HDAC1 and EGFR expression in **b** MBO fractions and **c** nuclear fractions of each cell line. Graphed values indicate mean ± standard error of the mean (SEM), *n* = 3. *p < 0.05; **p < 0.01; ***p < 0.001; ****p < 0.0001. **d** HDAC1 interaction with EGFR in the MBO and nuclear fractions of A549, PC-9, and H1975 cells was assessed. Quantification of EGFR interaction with HDAC1 in **e** MBO fractions and **f** nuclear fractions of each cell line. Graphed values indicate mean ± SEM, *n* = 3. ****p < 0.0001. **g** PC-9 cells were transfected with GFP-tagged HDAC1 expression plasmids, and were treated with 1 µM Gefitinib for 24 h. Cells were fixed with 4% paraformaldehyde and stained with Phalloidin and DAPI. The transfectants were examined for subcellular localization pattern of GFP-tagged HDAC1. The results are shown in the graph as the mean ± standard deviation (SD). The experiments were carried out in triplicate. For each experiment, >50 cells were examined. **h** The representative images are shown. Scale bar: 10 µm. **i** HEK293T cells were transfected with plasmids expressing Flag-tagged HDAC1 WT or Y72F mutant, and were exposed to 10 µM leptomycin B (LMB) for 4 h or 8 h. The expression of HDAC1 was examined and relatively quantified. **j** HEK293T cells were transfected with HA-tagged HDAC1 WT or Y72F mutant, and were exposed to 10 µM LMB for 4 h or 8 h. The expression of HDAC1 was assessed.

We explored in which cellular compartment HDAC1-EGFR interaction occurs. To this end, subcellular fractionation of A549, PC-9, and H1975 cells was performed to separate the cytosolic, membrane-bound organelle, and nuclear fractions^45^. HDAC1 and EGFR proteins were examined by immunoblotting (Fig. 5a) and their relative levels were quantified (Fig. 5b, c). In accordance with the literature, HDAC1 was detected in the nuclear fraction of each cell line, which includes the nuclear membrane. EGFR was also detected in the nuclear fraction at low levels. These fractions were subjected to immunoprecipitation with the anti-HDAC1 antibody to examine the HDAC1-EGFR interaction (Fig. 5d), and relative amounts of the interactions either in the membrane-bound organelle fraction (Fig. 5e) or in the nuclear fractions (Fig. 5f) were quantified. Notably, both HDAC1 and EGFR were present in the membrane-bound organelle fraction of each cell type. The HDAC1-EGFR interaction was detected in the membrane-bound organelle fraction of A549 cells, but was almost undetectable in PC-9 or H1975 cells, whereas it was consistently observed in the nuclear fractions of A549, PC-9 and H1975 cells. These results confirm the EGFR-HDAC1 interaction in lung adenocarcinoma cells, and indicate that the characteristics of their interaction can vary in different cell lines.

Although the HDAC1-EGFR interaction was predominantly observed in the nuclear fraction of PC-9 cells (Fig. 5d), the EGFR activity does affect the stability of HDAC1 protein likely in a manner dependent on ubiquitin-proteasome degradation pathway (Fig. 4c, d). Since ubiquitin-proteasome-dependent degradation is completed in the cytosol, we sought to dissect the potential relationship between the nuclear HDAC1-EGFR interaction and the cytosolic proteasomal degradation of HDAC1. To this end, we examined if the EGFR affects the export of HDAC1 from the nucleus. PC-9 cells were transfected with plasmid expressing GFP-tagged HDAC1, and were treated with Gefitinib or control. The subcellular localization of GFP-tagged HDAC1 in the transfected cells was observed (Fig. 5g, h). GFP-tagged HDAC1 was primarily localized in the nucleus of the transfected PC-9 cells, and EGFR activity seems to play a role in promoting the nuclear accumulation of HDAC1 protein. GFP-tagged HDAC1 could be localized to cytosol in ~13.6% of transfected cells in the control group. In contrast, ~79.4% Gefitinib-treated transfected cells showed cytosolic GFP-tagged HDAC1. It should be noted that the transfected cells have not been exposed to MG-132; considering the promotive effect of EGFR inhibition on HDAC1 degradation (Fig. 4a, d), the cytosolic accumulation of GFP-tagged HDAC1 caused by Gefitinib treatment is likely not due to the proteasome impairment, implying that the EGFR activity blocks the export of GFP-tagged HDAC1 from the nucleus.

We next asked if EGFR stabilizes HDAC1 protein by blocking its nuclear export. If such is the case, blockage of the nuclear export should attenuate the effect of EGFR inhibition on HDAC1 protein stability. We tested this hypothesis using the Y72F mutant HDAC1, which highly mimics the hypophosphorylation of HDAC1 and is less stable compared to the WT HDAC1. HEK293T cells were transfected with the plasmid expressing Flag-tagged HDAC1 WT or Y72F mutant, and were subjected to treatment with leptomycin B (LMB), a nuclear export inhibitor (CRM1 inhibitor) which can effectively block the export of HDAC1 from the nucleus^40^. The expression of Flag-tagged HDAC1 was examined by immunoblotting (Fig. 5i). The treatment with LMB resulted in an increase in the relative amount of Flag-tagged Y72F (to the WT) from 0.48 to 0.77. Similarly, when cells were transfected with HA-tagged HDAC1 expression plasmids and exposed to LMB, the relative amount of HA-tagged Y72F mutant increased from 0.53 to 0.81 (Fig. 5j). Together, these results suggest that active EGFR-mediated blockage of nuclear export of HDAC1 may contribute to its stability.

### Tyr72 supports the anti-apoptotic role of HDAC1

HDAC1 inhibition and EGFR inhibition have both been found to promote apoptosis of cancer cells^12,46,47^. Moreover, it has been determined that broad-spectrum HDAC inhibitors can synergize with EGFR inhibitors, leading to increased apoptosis in cancer cells and sensitization of resistant cells to EGFR inhibition^48,49^. To explore the role of the nonphosphorylatable Y72F mutant HDAC1 in regulating apoptosis in lung adenocarcinoma cells, PC-9 stable cell lines overexpressing WT or Y72F mutant HDAC1 in a control or *HDAC1*-knockdown background were established. PC-9 cells were first transduced with a retroviral control vector, WT HDAC1, or Y72F mutant HDAC1 and selected for positive clones. Each cell line was then transduced with either an shRNA control vector or an *HDAC1*-specific shRNA targeting the 3’UTR so as to only silence the expression of endogenous *HDAC1*. It should be noted that the strategy to first silence endogenous *HDAC1* and subsequently overexpress WT or mutant HDAC1 was unsuccessful due to the inability of *HDAC1*-knockdown cells to survive following the second transduction and selection process.

**Fig. 6:**
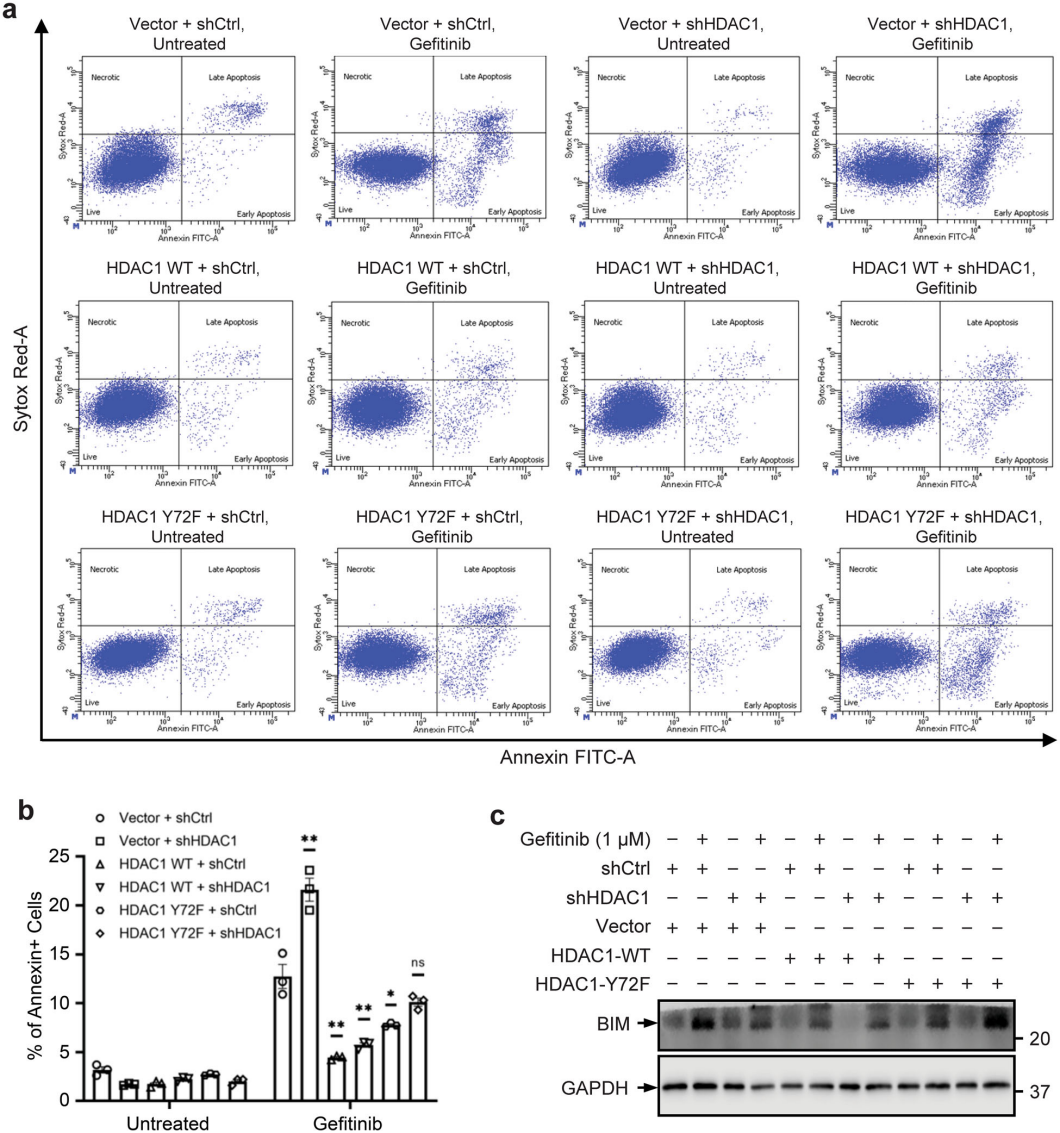
Role of HDAC1 Tyr72 in Gefitinib-mediated apoptosis. **a** PC-9 cells were treated with 1 μM Gefitinib for 24 h and Annexin+ cells were detected by flow cytometry analysis. Representative flow cytometry charts of untreated and Gefitinib-treated PC-9 cell lines stained with Annexin-FITC and Sytox Red. **b** Graphed values indicate mean ± SEM, *n* = 3. Significance of values was assessed relative to the Vector + shCtrl Gefitinib-treated condition. *p < 0.05; **p < 0.01. **c** BIM expression in PC-9 cell lines treated with 1 μM Gefitinib for 24 h.

To assess how the loss-of-phosphorylation mutation at Tyr72 affects the role of HDAC1 in apoptosis, cells were treated with 1 μM Gefitinib for 24 h and apoptosis was assessed by the percentage of Annexin V + cells (Fig. 6a, b). Gefitinib treatment induced apoptosis in each cell line, though to different extents. The greatest induction of apoptosis upon Gefitinib treatment was in the *HDAC1*-knockdown cells, consistent with literature evaluating the combined inhibition of EGFR and HDAC1. In contrast, WT HDAC1 overexpression in both the control and *HDAC1*-knockdown backgrounds promoted resistance against Gefitinib treatment, as indicated by the reduction in apoptotic cells relative to the other Gefitinib-treated cell lines. While overexpression of Y72F mutant HDAC1 also had a protective effect against EGFR activity inhibition, its extent of protection was reduced, as a higher percentage of apoptotic cells was observed in these conditions relative to cells overexpressing WT HDAC1. Additionally, following Gefitinib treatment, the extent of apoptosis in cells overexpressing Y72F mutant HDAC1 in the *HDAC1*-knockdown background was similar to that in control (Vector + shCtrl) cells. Furthermore, upregulation of the pro-apoptosis marker BIM has been observed upon EGFR and HDAC1 inhibition^12,46,47,50,51^. In each of the established PC-9 stable cell lines, the expression of BIM was increased upon Gefitinib treatment (Fig. 6c). However, the level of BIM was lower in cells overexpressing WT HDAC1 relative to other conditions. Overall, these results indicate that HDAC1 overexpression contributes to resistance to Gefitinib-induced apoptosis and suggest that the likely loss of phosphorylation at Tyr72 plays a role in apoptosis downstream of Gefitinib-mediated EGFR activity inhibition.

## Discussion

The role of HDAC1 in regulating processes such as cell cycle regulation, apoptosis, and DNA repair, among others, in cancer is well-established^5–7^. Given its extensive contribution to maintaining cell survival, HDAC1 is therefore an important target to eliminate cancer cells by apoptosis. However, while chemical inhibition of Class I HDACs, including HDAC1, has shown promise in the treatment of blood cancers, no such inhibitors have been approved for clinical use against solid tumors^52^. Thus, it is necessary to better understand HDAC1 regulation to identify new strategies by which to specifically and effectively target aberrations in its expression or activity in different tumor types.

The expression and activity of HDAC1 are tightly regulated by phosphorylation. Casein kinase 2 (CK2) phosphorylates HDAC1 at S421 and S423, which promotes its interactions with proteins in the CoREST, NuRD, and Sin3 corepressor complexes and its histone deacetylase activity^22^. HDAC1 phosphorylation at S421 and S423 was also related with the activity of p70 S6 kinase (S6K1) in breast cancer cells, occurring upon the activation of the phosphoinositide 3-kinase (PI3K)/ mammalian target of rapamycin (mTOR) pathway downstream of mitogenic stimulation. HDAC1 phosphorylation under this condition was associated with the deacetylation of the estrogen receptor α (*ERα*) promoter and reduction in its gene expression^20^. HDAC1 phosphorylation by Aurora kinases A and B at S406 during mitosis, however, reduces its histone deacetylase activity. This modification is transient in zebrafish, where HDAC1 phosphorylation results in the reduced deacetylation of genes relevant to the central nervous system that are involved in embryo development, and the basal state is necessary to maintain cell proliferation and zebrafish morphology^21^. Previously, the potential tyrosine phosphorylation of HDAC1 at several tyrosine residues (Tyr72, Tyr87, Tyr204, Tyr358) was detected in PC-9 cells^25^, but its biological function has not yet been explored further. The transient nature of tyrosine phosphorylation, its occurrence or augmentation under specific cellular conditions, and sensitivity of detection methods are possible explanations for the dearth of knowledge regarding this modification^4,53^. Given the roles of HDAC1 in different cell growth and survival mechanisms, we assessed the yet unevaluated possibility that tyrosine phosphorylation could regulate HDAC1 in NSCLC cells.

Using a general phosphotyrosine cocktail antibody, we detected that endogenous HDAC1 is indeed modified by tyrosine phosphorylation in lung adenocarcinoma cell lines. The level of HDAC1 tyrosine phosphorylation was notably higher in PC-9 cells, which express constitutively-active mutant EGFR, relative to WT EGFR-expressing A549 cells (Fig. 1a). It should be noted that, for this study, detection of HDAC1 tyrosine phosphorylation posed a challenge until large quantities of cells were collected for western blot analysis of immunoprecipitated HDAC1. Moreover, we pinpointed that Tyr72 residue is a critical determinant of the HDAC1 expression (Fig. 1c–g); loss-of-phosphorylation mutation of HDAC1 at Tyr72 led to a reduction in its protein expression. We further identified that HDAC1 robustly interacts with EGFR in lung adenocarcinoma cell lines, as well as HEK293T cells, by the high-confidence K-CLASP technique and co-immunoprecipitation. In line with this, we found that EGFR phosphorylates HDAC1 in vivo and in vitro. Inhibition of EGFR activity by the TKI Gefitinib, or by shRNA-mediated EGFR knockdown, reduced the tyrosine phosphorylation of HDAC1 to a level below detection (Fig. 3). Besides reducing its phosphorylation level, EGFR inhibition by Gefitinib, as well as by AG-1478, led to a reduction in the level of HDAC1 protein (Fig. 4a–c). In contrast, co-expression of WT EGFR, which corresponds to its constitutive activation by gene amplification^54^, resulted in a much higher increase in the protein levels of WT HDAC1, compared to that of the Y72F mutant (Fig. 4g), highly supporting that EGFR-mediated phosphorylation at Tyr72, rather than at other tyrosine residues, plays a determinant role in the regulation of HDAC1 protein stability. One of the next steps will be to develop a site-specific phosphorylation antibody to indicate with certainty that HDAC1 is indeed modified by phosphorylation at Tyr72 and further characterize the dynamics of this modification upon changes in EGFR activity.

Of note, Tyr72 is evolutionarily conserved over a range of species, suggesting that this residue may be of functional importance. This residue is also conserved in the other Class I members as Tyr73 in HDAC2, Tyr66 in HDAC3, and Tyr75 in HDAC8. Importantly, HDAC2 and HDAC3 both feature an EGFR kinase motif in the sequence spanning Tyr73 and Tyr66, respectively, and both of these residues were detected as phosphorylation sites under the same conditions as HDAC1 Tyr72^25,55^. We sought to test whether the phenotype observed in HDAC1 could be extended to the other Class I HDACs using a similar strategy as with HDAC1. However, unlike the Y72F mutation in HDAC1, the YF loss-of-phosphorylation mutation at the corresponding residues did not lead to a reduction in the expression of the other Class I HDACs (data not shown). These results suggest that the observed phenomenon is specific to HDAC1 and that the other Class I HDACs may be regulated differently if they are modified at this site.

Several studies have demonstrated different interactions between HDACs and EGFR. Notably, HDAC inhibition has been found to impact EGFR expression and activity. Treatment of colorectal cancer cells with the HDAC inhibitors Trichostatin A (TSA) or suberoylanilide hydroxamic acid (SAHA) was associated with reduced EGFR protein levels, for which HDACs 1, 2, and 3 were proposed to be responsible. HDAC3 was also identified to contribute to the transcriptional regulation of EGFR^56^. TSA treatment was also associated with reduced EGFR expression in MDA-MB-468 breast cancer and A431 epidermoid carcinoma cell lines, as well as head and neck squamous cell carcinoma (HNSCC) cells^56,57^. In HNSCC cells specifically, HDAC inhibition was related with the proteasomal degradation of EGFR^57^. HDAC inhibition by SAHA in estrogen receptor α (ER)-negative breast cancer cells has been associated with lower EGFR expression due to a reduction in its mRNA stability^58^. In contrast, HDAC6 promotes EGFR protein stability. However, EGFR phosphorylates HDAC6 to reduce its tubulin deacetylase activity, which in turn promotes EGFR trafficking leading to its degradation^59^. Still, little is known about the relationship between EGFR and HDAC1, specifically in lung cancer.

Here we found that HDAC1-EGFR interaction patterns change in lung adenocarcinoma cell lines with different EGFR backgrounds, albeit to varying extents. EGFR and HDAC1 were present in the membrane-bound organelle and nuclear fractions of lung adenocarcinoma cell lines. While their interaction could be detected in the nuclear fraction of each of the tested cell lines, there was a notable disparity in the membrane-bound organelle fraction, where their interaction was significantly higher in the WT EGFR-expressing A549 cells than in cell lines expressing EGFR activating mutations. These results also support the possibility that the status of EGFR may affect the dynamics of its relationship with HDAC1. The differences in the interactions between HDAC1 and either WT or mutant EGFR could be due to intricacies of EGFR activity, as the exon 19 deletion (in PC-9 cells), L858R mutation (in H1975 cells), and other mutations in its tyrosine kinase domain cause EGFR to remain in the constitutively-active conformation^60,61^. It is possible that the sustained activity of mutant EGFR may promote a relatively transient interaction such that it phosphorylates and readily dissociates from HDAC1, whereas the WT form may have a more stable interaction with HDAC1. These findings are also of particular interest since EGFR is classically considered a receptor tyrosine kinase that is localized at the cell membrane^37^, whereas it has been established that HDAC1 is a nuclear protein^1^. In recent years, there have been reports of the localization of EGFR in intracellular vesicles and the nucleus^42^. Several mechanisms of EGFR trafficking to the nucleus have been proposed and reviewed^62^. Nuclear EGFR has been found to phosphorylate nuclear substrates, such as histone H4, and regulate the transcription of genes^63,64^. In the present scenario, EGFR may similarly translocate to the nucleus to modify and stabilize HDAC1.

The ubiquitin-proteasome pathway plays an important role in regulating the turnover of many intracellular proteins, including HDAC1^33,34^. In this study, we found that Gefitinib treatment-induced reduction of HDAC1 protein could be, at least partially, rescued by proteasome inhibition (Fig. 4d). Moreover, our findings suggest that EGFR-mediated phosphorylation at Tyr72 protects HDAC1 from being ubiquitinated (Fig. 4e, f). The Tyr72 phosphorylation may directly or indirectly change the conformation of HDAC1 protein, in turn altering its interactions with ubiquitin-proteasome machineries. Specifically, MDM2 is reported to initiate HDAC1 K74 ubiquitination to promote HDAC1 degradation^34^. Thus, it is likely that Tyr72 (Y72) phosphorylation interferes with the K74 ubiquitination and degradation of HDAC1 due to the adjacent/close position of these two residues. In addition, we found that EGFR inhibition increased cytosolic sequestration of HDAC1 protein (Fig. 5g, h), and blocking the nuclear export effectively reduced the effect of Tyr72 phosphorylation on HDAC1 expression (Fig. 5i, j). Our findings clearly indicated an involvement of EGFR activity in regulating the nucleocytoplasmic shuttling of HDAC1, which contributes to the expression and stability of HDAC1. The export of HDAC1 is mediated by interaction with CRM1 and could be regulated by serine phosphorylation^40,65^. It is possible that Tyr72 phosphorylation may attenuate the HDAC1-CRM1 interaction, thus blocking its export from the nucleus. However, since the ubiquitination takes place in either nucleus or cytosol, and the ubiquitination state of proteins frequently impacts their nuclear export, the precise mechanism by which EGFR-mediated phosphorylation coordinates with nuclear export and ubiquitination to control the proteasomal degradation of HDAC1 remains to be further clarified.

EGFR inhibition by Gefitinib causes apoptosis in sensitive constitutively-active EGFR-mutant lung adenocarcinoma cells^66,67^. Furthermore, treatment of lung adenocarcinoma cells with broad-spectrum HDAC and Class I HDAC-specific inhibitors alone or in combination with an EGFR inhibitor induces anti-tumor effects, including apoptosis and a reduction in cell migration, angiogenesis, and proliferation, as well as re-sensitization of cells that were resistant to chemotherapy drugs or EGFR inhibition^18,48,49,68^. Here, whereas overexpression of WT HDAC1 promoted resistance to Gefitinib-induced apoptosis, the loss-of-phosphorylation Y72F mutation of HDAC1 reduced this effect and achieved approximately the same extent of apoptosis as control PC-9 cells treated with Gefitinib. Further, the reduction in BIM levels upon overexpression of WT HDAC1, but not of the Y72F mutant, are in line with studies showing that EGFR inhibition leads to increased BIM levels but lower levels of BIM are associated with resistance^54^. Thus, the phosphorylation of Tyr72 by EGFR not only is important for HDAC1 expression (Fig. 1), but also plays a role in regulating its anti-apoptotic function (Fig. 6).

**Fig. 7:**
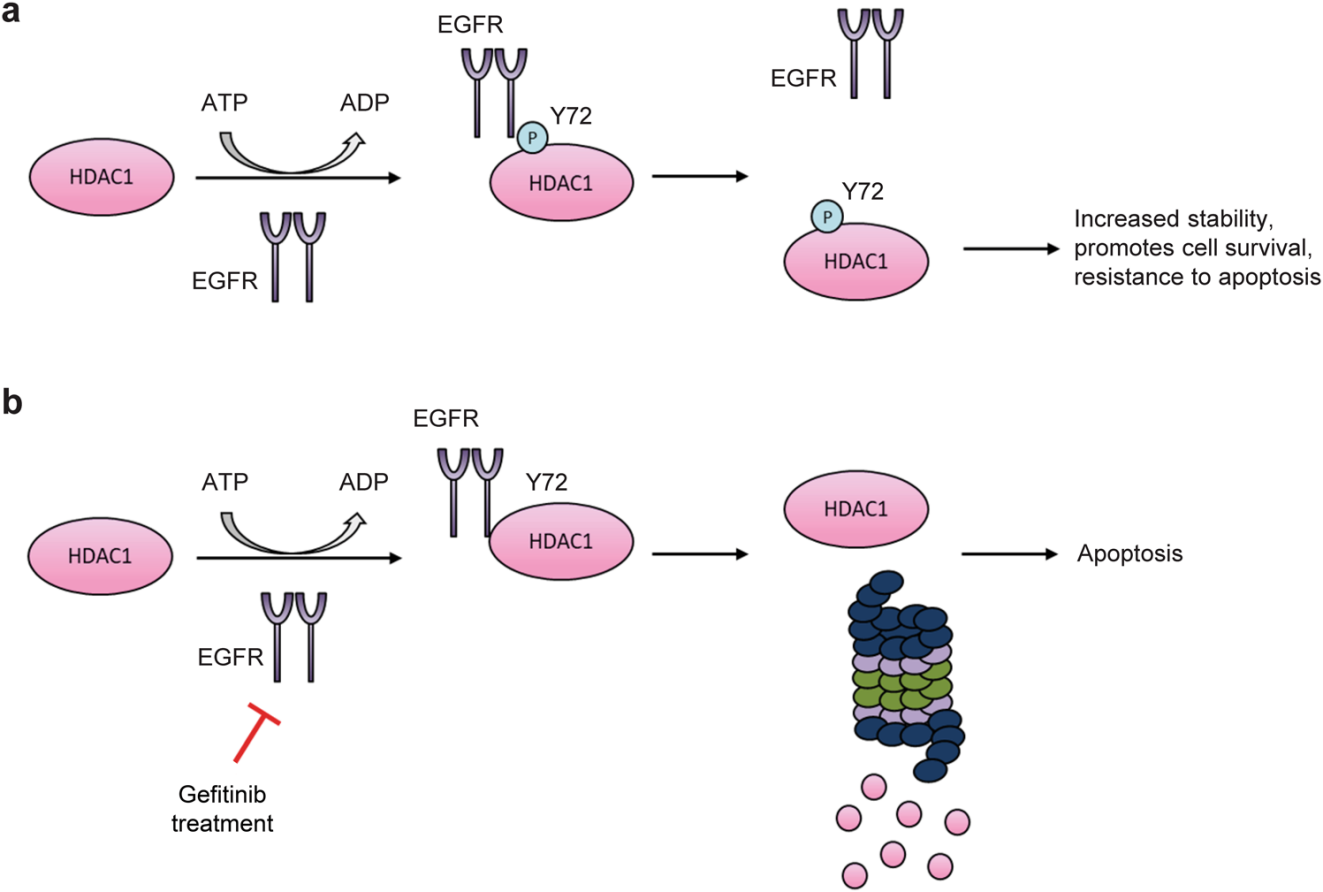
Model of the proposed role of HDAC1 Tyr72 phosphorylation in lung adenocarcinoma cells. **a** EGFR transiently interacts with HDAC1 and catalyzes its phosphorylation at Tyr72. Phosphorylation stabilizes HDAC1 and promotes its role in cell survival, where increased expression of HDAC1 contributes to resistance against apoptosis. **b** Gefitinib-mediated inhibition of EGFR tyrosine kinase activity inhibits HDAC1 phosphorylation, although HDAC1 and EGFR interaction is not inhibited. Subsequent proteasomal degradation of HDAC1 plays a role in Gefitinib-induced apoptosis of lung adenocarcinoma cells.

In summary, this study uncovered a previously uncharacterized interaction of EGFR with HDAC1 in lung adenocarcinoma cell lines. EGFR may phosphorylate HDAC1 at Tyr72 to stabilize HDAC1 by protecting it from ubiquitination, which in turn promotes cell survival and reduces apoptosis, whereas hypo-phosphorylation of HDAC1 due to the EGFR inhibition can reduce its protein stability and induce apoptosis (Fig. 7). The dynamics of EGFR activity and possible crosstalk between several possible PTMs, including other phosphorylation sites, on HDAC1 warrant further study to characterize the intricacies of HDAC1 regulation in EGFR-driven cancers.

## Materials and methods

### Antibodies and chemicals

Anti-HDAC1 antibody was previously generated in our laboratory^69^. Anti-FLAG M2 (F1804) and anti-β-actin (A1978) antibodies were purchased from Sigma-Aldrich (St. Louis, MO). Anti-glyceraldehyde-3-phosphate dehydrogenase (GAPDH, HRP-60004) antibody was from Proteintech (Rosemont, IL). Anti-Lamin A (sc-20680) and anti-HA tag (sc-805) antibodies were purchased from Santa Cruz Biotechnology (Dallas, TX). Anti-EGFR (A300-388A) antibody was from Bethyl Laboratories (Montgomery, TX). Anti-phospho-EGFR (Y1173) (AF1095) antibody was from R&D Systems (Minneapolis, MN). Anti-Bim (2876) and anti-Myc tag (9B11, 2276) antibodies were from Cell Signaling Technology (Danvers, MA). Phosphotyrosine antibody cocktail (13-6620) was from Invitrogen (Thermo Fisher Scientific, Waltham, MA). Mouse (NA931) and Rabbit (NA934) IgG HRP-linked antibodies were purchased from GE Healthcare (Chicago, IL).

Rhodamine Phalloidin (PHDR1) was from Cytoskeleton (Denver, CO). Epidermal Growth Factor (EGF; cat. 236-EG) was purchased from R&D Systems. Gefitinib (ZD1839) was from Cayman Chemical Company (Ann Arbor, MI). AG-1478 (A8357) and MG-132 (A2585) were from ApexBio (Houston, TX). Cycloheximide (C1988) and leptomycin B (L2913) were purchased from Sigma-Aldrich.

### Cell culture

HEK293T, GP2-293, and the NSCLC cell lines A549, PC-9, H1975, and H1299 were grown in Dulbecco’s modified Eagle medium (DMEM) supplemented with 10% heat-inactivated fetal bovine serum (FBS) and 1% penicillin-streptomycin at 37 °C in a humidified atmosphere with 5% CO_2_. Cells were from American Type Culture Collection (Manassas, VA).

### EGFR activation and inhibition

For EGFR activation, cells at 70% confluency were serum-starved for 16 h and were then stimulated by treatment with 20 ng/ml EGF for 30 min. For inhibition of EGFR activity, cells at 70% confluency were treated with the indicated dose of Gefitinib or AG-1478 in DMEM supplemented with 0.1% FBS for 24 h, after which cells were collected.

### Plasmids

Flag and GFP-tagged HDAC1 expression plasmids have been described^70^. HA and GST-tagged HDAC1 expression plasmids were generated by subcloning HDAC1 cDNA in-frame into pcDNA3.1-HA and pGEX4T-1 vector, respectively. Retroviral plasmids for HDAC1 expression were generated by subcloning HDAC1 cDNA into pBABE-neo vector. HDAC1 mutant expression plasmids were generated by PCR-based site-directed mutagenesis. Mutations were confirmed by sequencing. pCDNA6A-EGFR WT was a gift from Mien-Chie Hung (Addgene plasmid #42665; http://n2t.net/addgene:42665; RRID:Addgene_42665). pCDCN3-HA-Ub was a gift from Edward Yeh (Addgene plasmid #18712; http://n2t.net/addgene:18712; RRID:Addgene_18712). A His_6_-tagged ubiquitin expression plasmid was kindly provided by Jiandong Chen^35^. Lentiviral shRNA expression pLKO.1 plasmids targeting *EGFR* (shEGFR-1: TRCN0000295969, shEGFR-2: TRCN0000295968) and *HDAC1* (TRCN0000004814) were from Sigma-Aldrich (St. Louis, MO). pLKO.1-TRC control was from Addgene (Cambridge, MA).

### Transfection, virus preparation and transduction

Transfections were performed using polyethylenimine. Lentivirus was prepared by transfecting HEK293T cells with pLKO.1-TRC control or pLKO.1 shRNA plasmids to specifically target *EGFR* or *HDAC1*, in combination with viral packaging vectors. To establish the stable knockdown, cells were transduced with lentivirus and selected with puromycin. Retrovirus was prepared by transfecting GP2-293 cells with pBABE-neo vector or pBABE-neo plasmids expressing either WT HDAC1 or Y72F mutant together with viral packaging vectors. PC-9 cells were first transduced with retrovirus and selected with neomycin, and were then further transduced with either pLKO.1-TRC control or pLKO.1 shRNA plasmid targeting *HDAC1* and selected with puromycin.

### Whole cell lysate extraction and subcellular fractionation

To collect whole cell lysate, cells were lysed in RIPA buffer (25 mM Tris-HCl [pH 7.4], 0.1% SDS, 1% NP-40, 0.5% sodium deoxycholate, 150 mM NaCl, protease inhibitor cocktail, 1 mM Na_3_VO_4_, and 1 mM NaF). For subcellular fractionation, cells were trypsinized and centrifuged to collect the cell pellet. The cell pellet was incubated with Lysis Buffer A (50 mM HEPES [pH 7.4], 150 mM NaCl, 1 M hexylene glycol, 25 μg/mL digitonin, protease inhibitor cocktail, 1 mM Na_3_VO_4_, and 1 mM NaF) on ice for 10 min to extract the cytosolic proteins. The cell pellet was washed with Lysis Buffer A and was then incubated with Lysis Buffer B (50 mM HEPES [pH 7.4], 150 mM NaCl, 1 M hexylene glycol, 1% v/v Igepal, protease inhibitor cocktail, 1 mM Na_3_VO_4_, and 1 mM NaF) on ice for 30 min to extract the membrane-bound proteins. The cell pellet was then washed twice with Lysis Buffer B, after which it was incubated with Lysis Buffer C (50 mM HEPES [pH 7.4], 150 mM NaCl, 1 M hexylene glycol, 0.5% w/v sodium deoxycholate, 0.1% w/v sodium dodecyl sulfate, protease inhibitor cocktail, 1 mM Na_3_VO_4_, and 1 mM NaF) and benzonase to extract the nuclear proteins.

### Immunoprecipitation and immunoblotting

Whole cell lysate or subcellular fraction samples were pre-cleared using protein A/G agarose resin. The pre-cleared samples were then incubated overnight with the indicated antibodies and protein A/G agarose resin. Beads were washed five times and immunocomplexes were eluted with 0.2 M glycine (pH 2.3). For immunoblotting, samples were resolved by SDS-PAGE and then transferred to a nitrocellulose membrane. The membranes were incubated in blocking buffer (5% bovine serum albumin [BSA] in PBS-T or TBS-T) for 1 h at room temperature, and then incubated with primary antibodies in blocking buffer overnight at 4 °C, followed by incubation with secondary antibodies at room temperature for 2 h. ECL chemiluminescence detection reagents (Thermo Fisher Scientific) were used for imaging and bands were quantified by Image Studio™ Lite software (LI-COR Biosciences, Lincoln, NE).

### Flow cytometry analysis

For analysis of apoptosis by Annexin V, PC-9 cell lines were washed with HBSS and detached by trypsinization. DMEM was added to the cells and the cells were incubated at 37 °C for 30 min with occasional shaking to allow the cell membranes to recover from the trypsin treatment while keeping the cells detached. Following collection, a single cell suspension of 1 × 10^6^ cells/ml was prepared in Annexin V Binding Buffer (422201, Biolegend, San Diego, CA). While protected from light, samples were incubated first with FITC-Annexin V (640906, Biolegend) for 15 min at room temperature and then with Sytox Red dead cell stain (50-112-1578, Fisher Scientific) for an additional 15 min at room temperature. The samples were subsequently analyzed by flow cytometry using the 3-laser BD Celesta Analyzer. Analysis was performed by FCS Express.

### Kinase-catalyzed crosslinking and streptavidin purification (K-CLASP)

K-CLASP was performed as described previously^26^, except using A549 lysates (500 μg) and WT HDAC1 (Biotin-KYHSDDYIKFLRSI, 1 μM) and HDAC1 Y72A mutant (Biotin-KYHSDDAIKFLRSI, 1 μM) peptides that were purchased from GenScript (Piscataway, NJ). After LC-MS/MS analysis with label-free quantitation using MaxQuant^71^, fold enrichment values were calculated for each protein by dividing the intensity observed in the WT HDAC1 peptide crosslinked sample (with ATP-arylazide and UV) by the intensity of that same protein in 1) the WT peptide with ATP-arylazide and no UV, 2) the WT peptide with ATP and UV, and 3) the mutant peptide with ATP-arylazide and UV negative control samples. If all fold enrichment values of a protein were >1.5, the protein was selected as a possible hit. Cytoscape 3.7.1 (ref. ^72^) and GeneMANIA^73^ were used to perform interactome analysis.

### Statistical analyses

Statistical analyses were performed by unpaired *t*-test for comparison between two groups and repeated measures one-way ANOVA or two-way ANOVA for comparison of multiple treatment groups or conditions. Tests were two-sided, assuming a normal distribution, and three biological replicates (*n* = 3) were analyzed for each experiment unless otherwise indicated. GraphPad Prism software was used to perform the analyses and graph results. Graphs present the mean ± standard error of the mean (SEM) or standard deviation (SD), as indicated, with individual data points marked. A *p*-value of less than 0.05 is considered statistically significant. Significance is denoted as follows: *p < 0.05; **p < 0.01; ***p < 0.001; ****p < 0.0001.

## Supplementary information

Supplemental Tables

Supplemental Table Legends

## Data Availability

The datasets used and/or analyzed during the current study are available from the corresponding author on reasonable request.
