# Supplementary material for: EGFR phosphorylates HDAC1 to regulate its expression and anti-apoptotic function
Source: Cell Death Dis. 2021 May 11;12(5):469. doi: 10.1038/s41419-021-03697-6 (PMC8113371; doi:10.1038/s41419-021-03697-6)
Supplement: Supplementary file 2 — Supplemental Table Legends [file 41419_2021_3697_MOESM2_ESM.pdf]

Table Legends

Table S1: K-CLASP raw data

Table S4: The HDAC1 K-CLASP hit proteins
